# Diagnostic Vaccination in Clinical Practice

**DOI:** 10.3389/fimmu.2021.717873

**Published:** 2021-09-30

**Authors:** Anette Tarp Hansen, Anna Söderström, Charlotte Sværke Jørgensen, Carsten Schade Larsen, Mikkel Steen Petersen, Jens Magnus Bernth Jensen

**Affiliations:** ^1^ Department of Clinical Epidemiology, Aarhus University Hospital, Aarhus, Denmark; ^2^ Department of Clinical Immunology, Aarhus University Hospital, Aarhus, Denmark; ^3^ Department of Clinical Immunology and Transfusion Medicine, Karolinska University Hospital, Stockholm, Sweden; ^4^ Statens Serum Institut, Virus and Microbiological Special Diagnostics, Copenhagen, Denmark; ^5^ Department of Infectious Diseases, Aarhus University Hospital, Aarhus, Denmark

**Keywords:** diagnostic vaccination, primary immunodeficiency, antibody deficiency, vaccination, pneumococcal vaccines, z-score, clinical guidelines

## Abstract

Testing the antibody response to vaccination (diagnostic vaccination) is crucial in the clinical evaluation of primary immunodeficiency diseases. Guidelines from the American Academy of Allergy, Asthma & Immunology (AAAAI) provide detailed recommendations for diagnostic vaccination with pure pneumococcal polysaccharide vaccines (PPV). However, the degree of compliance with these guidelines and the utility of the guidelines in actual practice are undescribed. To address this, we systematically evaluated diagnostic vaccination in adult patients with suspected primary immunodeficiency diseases in a single tertiary center from 2011 to 2016 (*n* = 229). We found that full compliance with the AAAAI guidelines was achieved for only 39 patients (17%), suggesting that the guidelines are not easy to follow. Worse, interpretation according to the guidelines was heavily influenced by which serotype-specific antibodies that were used for the evaluation. We found that the arbitrary choices of serotype-specific antibodies could change the fraction of patients deemed to have ‘adequate immunity’ by a factor of four, exposing an inherent flaw in the guidelines. The flaw relates to dichotomous principles for data interpretation under the AAAAI guidelines. We therefore propose a revised protocol for diagnostic vaccination limited to PPV vaccination, subsequent antibody measurements, and data interpretation using Z-scores. The Z-score compiles multiple individual antibody levels, adjusted for different weighting, into one single continuous variable for each patient. In contrast to interpretation according to the AAAAI guidelines, the Z-scores were robust to variations in the choice of serotype-specific antibodies used for interpretation. Moreover, Z-scores revealed reduced immunity after vaccination in the patients with recurrent pneumonia (a typical symptom of antibody deficiency) compared with control patients. Assessment according to the AAAAI guidelines failed to detect this difference. We conclude that our simplified protocol and interpretation with Z-scores provides more robust clinical results and may enhance the value of diagnostic vaccination.

## Introduction

Test of antibody responses to vaccination (diagnostic vaccination) is pivotal in clinical evaluation of patients with suspected antibody deficiency (1–4). A typical symptom of antibody deficiency is recurrent airway infections, although additional infectious disease susceptibilities and comorbidities can be present (5, 6). Assessment of antibody competence is therefore a general recommendation for patients with suspected primary immunodeficiency diseases (7). Although diagnostic vaccination is widely used, the details of the procedure vary (8–11).

Diagnostic vaccination entails measurement of vaccine-specific serum antibodies before and after vaccination. Unconjugated 23-valent pneumococcal capsular-polysaccharide vaccines (PPV) are often used for this purpose. Detailed guidelines for the use of PPV in diagnostic vaccination were proposed in 2012 by the *American Academy of Allergy, Asthma & Immunology* (AAAAI) (7). These guidelines are based on several key concepts (1, 7, 12). First, serum levels of individual serotype-specific antibodies should be quantified before vaccination and four to eight weeks after vaccination. Second, dichotomous principles are recommended for data interpretation: i) antibody levels of 1.3 mg/L or higher are considered ‘protective’ against a given serotype and ii) adequate antibody immunity in adults requires ‘protective’ levels for at least 70% of the tested serotype-specific antibodies (7). Crucially, the exact number of serotype-specific antibodies for assessment and their serotype specificities are not defined. However, it is implicit in the guidelines that multiple different serotype-specific antibodies should be tested (7).

**Figure 1:**
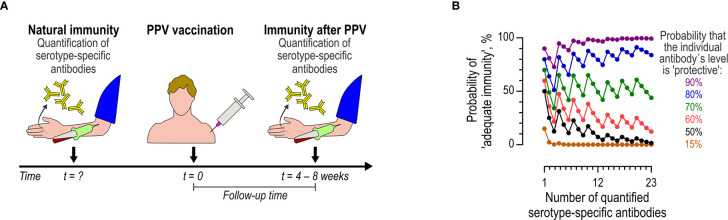
Diagnostic vaccination according to the AAAAI guidelines. **(A)** Flow of events. The preexisting immunity (natural immunity), represented by the levels of multiple (undefined number) serotype-specific antibodies, is determined at an undefined time-point before vaccination (t = ?). Later, PPV is administered (t = 0). The immunity after PPV is assayed four to eight weeks later by quantifying the same serotype-specific antibodies. **(B)** Model showing the theoretical probability of achieving adequate immunity (y-axis, left) according to the AAAAI guidelines (i.e., the probability of at least 70% of serotype-specific antibodies reaching levels of at least 1.3 mg/L) as a function of the number (x-axis) of tested serotype-specific antibodies. The colored curves represent different probabilities of an individual antibody being classified as ‘protective’ (i.e., a level of at least 1.3 mg/L). The probability of achieving ‘adequate immunity’ follows the binomial distribution, under the simplifying assumption that the individual serotype-specific antibodies in a given panel have equal likelihoods of being at the ‘protective’ level (although this will rarely be the case, the simplification nonetheless serves to illustrate the underlying problem).

Compliance with the AAAAI guidelines thus requires several correctly timed actions: two blood samplings, vaccination with the proper vaccine, measurements of antibody levels using an appropriate assay, and interpretation of immune status according to complicated rules (**Figure 1A**). We hypothesized that strict adherence to the AAAAI guidelines will often fail in actual clinical practice.

The recommended dichotomous approach to the interpretation of diagnostic vaccination is problematic. Such dichotomization introduces a complex, non-monotonic relationship between the probability of qualifying for adequate immunity and the number of tested serotype-specific antibodies (**Figure 1B**) (10). For example: when measuring seven, eight, or nine antibodies, the immunity is deemed ‘inadequate’ according to the guidelines if at least three antibodies are below the limit of 1.3 mg/L (because fewer than 70% of the antibodies will be ‘protective’). However, the probability that at least three antibodies are below 1.3 mg/L obviously increases with the number of antibodies tested. The probability of concluding ‘inadequate immunity’ in a patient is thus more likely when testing nine antibodies than when testing seven antibodies. A similar principle applies when more antibodies are tested (**Figure 1B**). Another weakness is that different serotype-specific antibodies do not have equal probabilities of reaching a level of at least 1.3 mg/L. In patients with suspected immunodeficiency disease, the mean levels differ for different serotype-specific antibodies (13–15). A limit of 1.3 mg/L regardless of specificity is thus somewhat arbitrary (16) and therefore not necessarily optimal. However, these factors ultimately decide the outcome of diagnostic vaccination, and therefore the clinical evaluation of the individual patient. Moreover, the dichotomous principles hinder comparison of patient cohorts, unless an identical panel of serotype-specific antibodies (and assays) are used.

**Figure 2:**
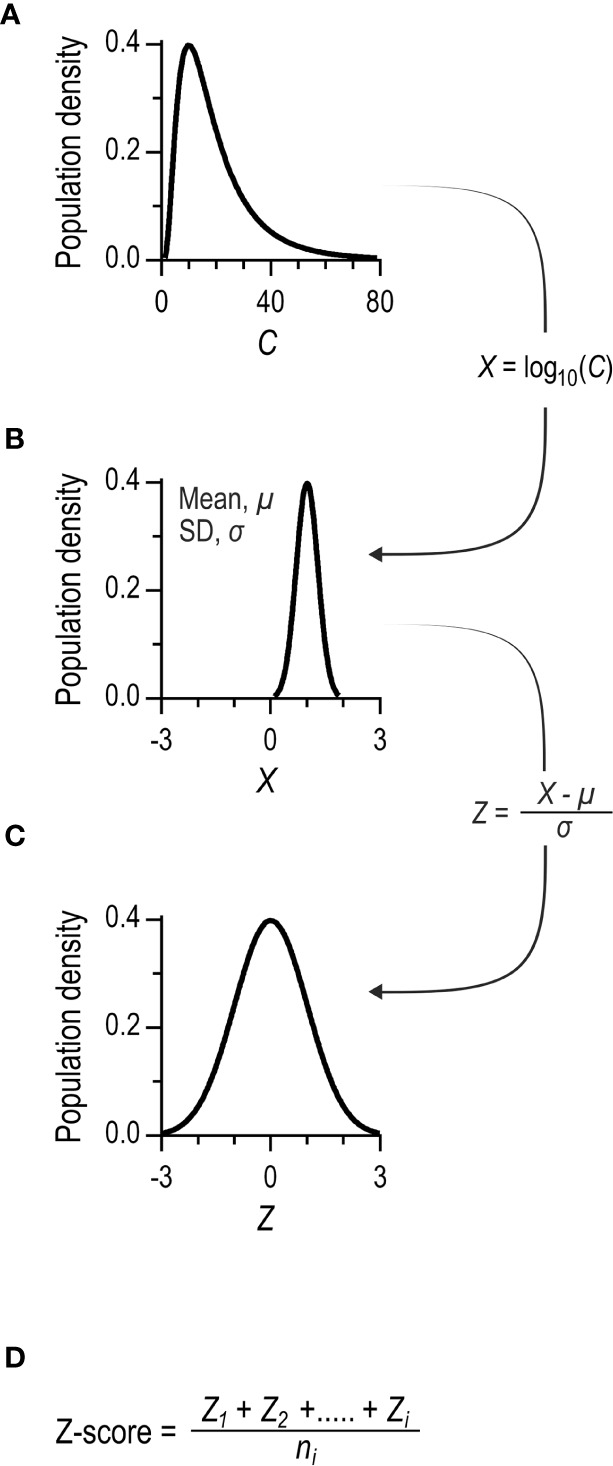
Principles for calculating Z-scores. **(A)** The distribution of serum concentrations of a serotype-specific antibody in a population. The distribution is typically left-skewed. **(B)** Log_10_ transformation of data results in Gaussian distribution. **(C)** The individual concentrations are further transformed to standard normal distributions. This is achieved by subtracting the mean and dividing by the standard deviation of the population dataset. The final parameter is dimensionless, and the population data set has a mean of 0 and a standard deviation of 1. **(D)** The Z-score is calculated for each patient as the mean of the standard normal deviations of the individual antibody levels. The Z-score´s standard deviation tends to decrease with an increasing number of distinct serotype-specific antibody levels, owing to mutual correlations (10). To promote comparability of cohorts tested with different number of measured serotype-specific antibody levels, the Z-score is normalized by the standard deviation of the population dataset.

Diagnostic vaccination using *continuous* variables for interpretation is more attractive from a theoretical standpoint (10). We have proposed using the Z-score, which is more robust than the dichotomous assessment to both the number of antibodies and their serotype-specificities (10). The Z-score is based on standard normal deviations of the individual serotype-specific antibodies (**Figure 2**). Because individual standard normal deviations are compiled by a simple mean, the complex relationship between the number of antibodies and the outcome (inherent in the dichotomous approach) is eliminated. A direct comparison of the outcomes of diagnostic vaccination using Z-score and the dichotomous principles is hitherto unreported for patients with suspected primary immunodeficiency diseases.

Our aims were thus i) to evaluate the degree of compliance with AAAAI guidelines for diagnostic vaccination in a tertiary center for primary immunodeficiency diseases and ii) to compare the outcome of diagnostic vaccination based on AAAAI guidelines to that based on Z-scores.

## Material and Methods

### Patients

**Figure 3:**
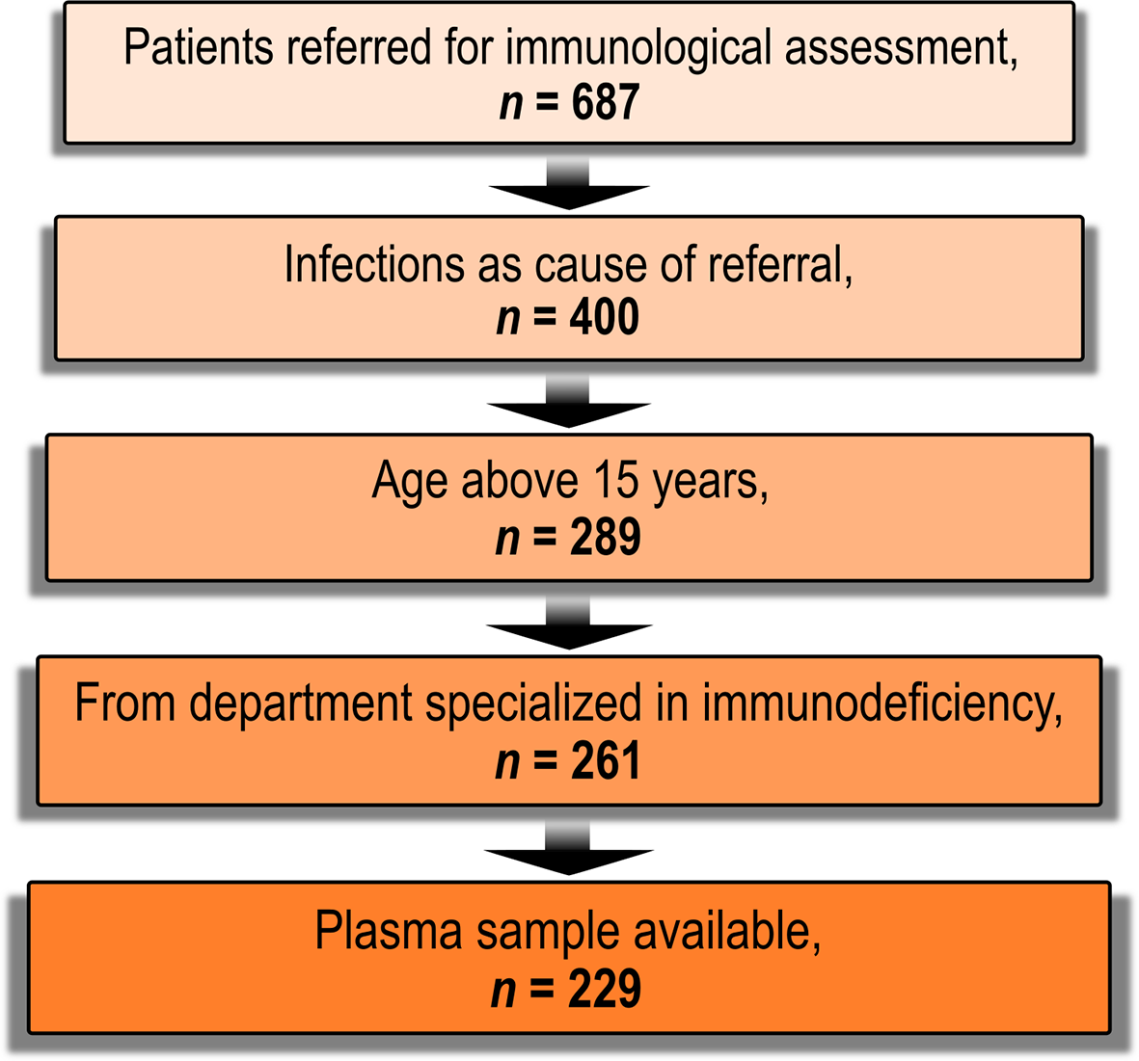
Flow-chart of patient enrolment. Candidates eligible for inclusion were patients referred for advanced laboratory assessment of immunodeficiency at the department of Clinical Immunology, Aarhus University Hospital, Denmark between 12 May, 2011 and 18 August, 2016 (*n* = 687). Patients without an increased susceptibility to infections were excluded (*n* = 287). Patients younger than 16 years were excluded (*n* = 111) as diagnostic vaccination is not local practice in these patients. We also excluded patients who were referred from departments not specialized in immunodeficiency (*n* = 28). Patients without a previously archived plasma sample were also excluded (*n* = 32).

Eligible patients were referred to advanced laboratory evaluation for immunodeficiency at the Department of Clinical Immunology, Aarhus University Hospital, Denmark over a five-year period (from May 2011 to August 2016, *n* = 687). Only patients referred from the Department of Infectious Diseases, Aarhus University Hospital, Denmark were included in the final cohort (*n* = 229). In Denmark, diagnostics and treatment of immunodeficiency is part of the general healthcare freely available to all citizens. The Department of Infectious Diseases, Aarhus University Hospital, Denmark is the specialized clinical center covering all adults with primary immunodeficiency diseases living in the Central Denmark Region (1.3 million inhabitants). All included patients were adults suspected of primary immunodeficiency disease by experienced infectious disease clinicians. We categorized patients into infection profiles based on referral data. The patient population consisted of both patients with normal immunoglobulin concentrations and patients with reduced immunoglobulin concentrations. This information was not systematically available for the present study. The patients represented the majority of the patients with idiopathic infections in a recent study (17). **Figure 3** shows a flow chart of the establishment of the final cohort. The predominant reason for exclusion was a lack of increased susceptibility to infections (*n* = 287).

For the audit, we obtained data on administered pneumococcal vaccines and IgG substitution from medical records. The study was conducted under the approval of the *Ethics Committee in Central Denmark Region* (reference number 1-10-72-127-12), and the Danish Data Protection Agency (reference number 1-16-02-40-12/2007-58-0010) in accordance with Danish legislation.

### Antibody Measurements

Evaluation of anti-pneumococcal antibody levels was part of the routine clinical practice at Department of Infectious Diseases, Aarhus University Hospital. Clinicians could choose between qualitative or quantitative antibody assays. For either assay, the concentration of specific IgG antibodies against 12 pneumococcal capsules (serotypes 1, 3, 4, 5, 6B, 7F, 9V, 14, 18C, 19A, 19F, and 23F) were determined in serum samples by an in-house Luminex-based method described by Lal et al. (18). Concentrations (in mg/L) were measured for each of the 12 measured serotype-specific antibodies. For quantitative assays, the concentrations were reported individually for the serotype-specific antibodies, as required for interpretation with the AAAAI guidelines. Qualitative assays were reported as ‘protective immunity’ when the geometrical mean of the 12 individual serotype-specific antibodies was at least 1 mg/L (the levels of individual serotype-specific antibodies were not reported); however, such data cannot be interpreted under the AAAAI guidelines. In Denmark, medical data are linked to the individual patient *via* the national Danish Personal Identification Number system. Using the identification numbers of the patients in the final cohort, we retrieved complete data on all measured anti-pneumococcal antibodies with the multiplex assay (preceding April 18, 2018). This also included quantitative data of measurements originally requested as qualitative by the clinicians.

### Assessment of Antibody Levels

We excluded measurements from patients who i) had received IgG replacement therapy within the previous six months, or ii) had a history of any pneumococcal vaccination before the study period, or iii) were previously vaccinated with protein-conjugated pneumococcal vaccines during the study period. Conjugate vaccines elicits an antibody response by different mechanisms than do natural infection or PPV vaccination (19), which can affect the response to subsequent vaccination with PPV (1, 7). Each of the 12 serotype-specific antibodies were quantifiable in all available measurements.

Natural immunity was defined as the serotype-specific antibodies that pre-existed before PPV vaccination. For patients with several available antibody measurements, we used the following rules to include a single measurement per patient only: patients who did not receive PPV during the study period had results from their first measurement included; and patients who received PPV during the study period had their results from the last measurement before PPV included. In the subset of patients for investigations of natural immunity, we also included the qualitative data from patients where the clinicians had requested qualitative measurements. The total subset comprised 154 patients.

Immunity after PPV. For patients with several antibody measurements, we selected the first measurement that occurred between four weeks and eight weeks after their PPV vaccination [(in compliance with the AAAAI guidelines (1, 7, 12)]. For patients who only had antibody measurements outside this interval, we included the antibody measurement closest to this interval. In the subset of patients for investigations of immunity after PPV vaccination, we also included the qualitative data from patients where the clinicians had requested qualitative measurements. The total subset comprised 98 patients.

Assessment by dichotomous principles. ‘Protective level’ for individual serotype-specific antibodies was defined as at least 1.3 mg/L in agreement with AAAAI guidelines (1, 7, 12). ‘Adequate immunity’ was defined as at least 70% of the interpreted serotype-specific antibodies having ‘protective levels’.

Z-scores were calculated as previously described (10). The principles are summarized in **Figure 2**. Briefly, the levels of individual serotype-specific antibodies were transformed to standard normal distributions. For each patient, the Z-score was the average of the standard normal deviation of each of the twelve individual antibodies. A Z-score (also called a standard score thus represents how many standard deviations a raw score is from the population mean.

### Statistics

We estimated 95%-confidence intervals (reported in square brackets) for effect sizes using Estimation Statistics (www.estimationstats.com) (20) and for means using t-distributions (continuous variables) or exact binomial statistics (dichotomous variables). STATA 11 (StataCorp LP, TX, USA) was used for data analysis other than estimations of effect sizes. Given the exploratory nature of our study, we refrained from making corrections for multiple comparisons (to limit risk of type II errors). To limit the risk of type I errors, we minimized the number of comparisons to those deemed strictly relevant. When more than two groups were available for comparisons, we therefore defined one shared control group. Graphs were made in GraphPad PRISM v. 6.07 (GraphPad Software, CA, USA). The level of significance was defined as 0.05.

## Results

### Study Population

The total cohort comprised 229 patients (**Figure 3**). Recurrent respiratory tract infections (a cardinal sign of antibody deficiency) was the predominant type of infection in 142 patients. These patients were subdivided into those with increased tendency to lower-respiratory tract infections (‘LRTI’, *n* = 114) and those with increased tendency to upper-respiratory infections without increased tendency to lower airway infections (‘URTI’, *n* = 28). The remaining 87 patients, labeled ‘control’, suffered from other types of infections (**Table 1**) that did not elicit suspicion of antibody deficiency. In the final cohort, 73% were female. The median age was 50 years (range 16 to 83 yrs.). Patients in the LRTI group were on average 11 [6; 15] years older than controls. The age of the patients in the URTI group was comparable to that of the control group.

**Table 1:** Patients categorized by their dominating type of infections.

	Case patients				Control patients										All patients	
	LRTI		URTI		Abscesses		Viral		Fungal		Invasive bacterial		Other			
Number	114		28		39		27		6		5		10		229	
Female, *n* (%)	87	(76)	23	(82)	27	(69)	20	(74)	2	(33)	4	(80)	5	(50)	168	(73)
Median age, yrs. (range)	57	(18–83)	45	(21–76)	39	(22–76)	43	(16–68)	55	(45–63)	40	(21–63)	56	(20–67)	50	(16–83)

LRTI: Patients with reported increased tendency to lower-respiratory tract infections.

URTI: Patients with reported increased tendency to upper-respiratory tract infections without reported increased tendency to lower-airway infections.

### Compliance With the Guidelines for Diagnostic Vaccination

For our evaluation of compliance with the AAAAI guidelines in clinical practice, we categorized the patients as those with i) failed initiation, ii) failed procedure, and iii) completed procedure (**Table 2**).

**Table 2:** Compliance with the AAAAI guidelines for diagnostic vaccination.

				Case patients				Control patients										All patients,	
				LRTI, *n* = 114		URTI, *n* = 28		Abscesses, *n* = 39		Viral, *n* = 27		Fungal, *n* = 6		Invasive bacterial,* n* = 5		Other, *n* = 10		*n* = 229	
**Failed initiation** (serotype-specific antibodies not measured)**, *n* (%)**				**6**	**(5.3)**	**1**	**(3.6)**	**13**	**(33)**	**16**	**(59)**	**2**	**(33)**	**4**	**(80)**	**5**	**(50)**	**47**	**(21)**
**Failed procedure, *n* (%)**				**86**	**(75)**	**20**	**(71)**	**20**	**(51)**	**9**	**(33)**	**3**	**(50)**	**1**	**(20)**	**4**	**(40)**	**143**	**(62)**
	Serotype-specific antibody measurements:																		
		Never quantitative		22	(19)	1	(3.6)	3	(7.7)	7	(26)	1	(17)	0		1	(10)	35	(15)
		Lacking before vaccination		16	(14)	6	(21)	3	(7.7)	0		0		0		1	(10)	26	(11)
		Only qualitatively before vaccination		5	(4.4)	2	(7.1)	1	(2.6)	0		0		1	(20)	2	(20)	11	(4.8)
	Natural immunity inadequate but:																		
		PPV not administered		14	(12)	9	(32)	9	(23)	2	(7.4)	2	(33)	0		0		36	(16)
		PPV administered but follow-up antibody measurements:																	
			Lacking	4	(3.5)	1	(3.6)	1	(2.6)	0		0		0		0		6	(2.6)
			Qualitative	14	(12)	0		1	(2.6)	0		0		0		0		15	(6.6)
			Quantitative but before week 4	1	(0.88)	0		0		0		0		0		0		1	(0.44)
			Quantitative but after week 8	7	(6.1)	0		1	(2.6)	0		0		0		0		8	(3.5)
	Date of PPV vaccination uncertain			3	(2.6)	0		0		0		0		0		0		3	(1.3)
	Previous administered conjugate pneumococcal vaccine			0		1	(3.6)	1	(2.6)	0		0		0		0		2	(0.87)
**Completed procedure, *n* (%)**				**22**	**(19)**	**7**	**(25)**	**6**	**(15)**	**2**	**(7.4)**	**1**	**(17)**	**0**		**1**	**(10)**	**39**	**(17)**
	Adequate natural immunity, PPV not administered			1	(0.9)	2	(7.1)	3	(7.7)	1	(3.7)	1	(17)	0		0		8	(3.5)
	Adequate natural immunity, PPV administered (superfluous)			0		1	(3.6)	0		1	(3.7)	0		0		0		2	(0.87)
	Inadequate natural immunity, PPV administered and follow-up with quantitative antibody measurements			21	(18)	4	(14)	3	(7.7)	0		0		0		1	(10)	29	(13)

Each participant was assigned to the first correct category in the left column (top-to-bottom). Adequate natural immunity was defined as ≥ 70% of measured levels of serotype-specific antibodies ≥ 1.3 mg/L.

Categories of compliance are indicated in bold.

Failed initiation was concluded for patients where anti-pneumococcal antibodies were never measured. This applied to 47 cases (21%). The finding was more common in the control group, 46%, compared with the LRTI group, 5.3% (difference -41% [-52%; -29%], i.e., 9-fold difference), and 3.6% in the URTI group (difference -42% [-52%; -25%], i.e., 13-fold difference).

Failed procedure was concluded for patients where the procedure had been commenced, (i.e., the antibodies had been quantified) but the available data were insufficient for interpretation under AAAAI guidelines. This applied to most patients (62%). Of the commenced procedures (*n* = 182), no group difference was found for failure frequency: 79% in the control group, 80% in the LRTI group (difference 0.91% [-13%; 16%]), and 74% in the URTI group (difference -4.7% [-27%; 14%]). The reason for failed procedure differed. The most common cause was a request of qualitative antibody assay instead of the required quantitative assay. This accounted for 43% of all failed procedures. We compared the outcome of the available qualitative assay (‘protective immunity’ defined as a geometrical mean of individual serotype-specific antibodies of at least 1 mg/L) with outcomes based on the AAAAI guidelines. The former concluded four times as many of the evaluations as ‘protective immunity’ than the latter (**Figure S1**). The second most frequent cause of failed procedure was a lack of PPV vaccination despite proven inadequate natural immunity. This explained 25% of the failed procedures. The remaining causes of failed procedures are given in **Table 2**.

Completed diagnostic vaccination was concluded for patients with i) documented adequate natural immunity or ii) documented inadequate natural immunity followed by PPV vaccination and quantification of serotype-specific antibodies four to eight weeks later. This applied to 39 patients (17%) only. Of the commenced procedures (*n* = 182), 21% were completed overall. No group difference was found: 21% in the control group, 20% in the LRTI group (difference -0.91% [-16%; 12%]), and 26% in the URTI group (difference 4.7% [-14%; 26%]).

In conclusion, compliance with the AAAAI guidelines seems difficult to accomplish in routine settings. Different meticulousness among physicians could be an important factor. In our setting, two clinicians were responsible for 93% of the referred patients. Clinician A referred 115 of the patients and completed the procedure for 23% of these. Clinician B referred 97 of the patients and completed the procedure for 11% of these (i.e., two-fold lower completion frequency). Thus, even among experienced clinicians, the chance of completing diagnostic vaccination according to the AAAAI guidelines varies markedly.

### Levels of Serotype-Specific Antibodies in the Cohort

We claim that the outcome of diagnostic vaccination conducted under AAAAI principles is influenced by the choice of antibody specificities for evaluation. The various antibodies cannot be expected to have the same probability of achieving a concentration of at least 1.3 mg/L. We examined this in details for the cohort.

**Figure 4:**
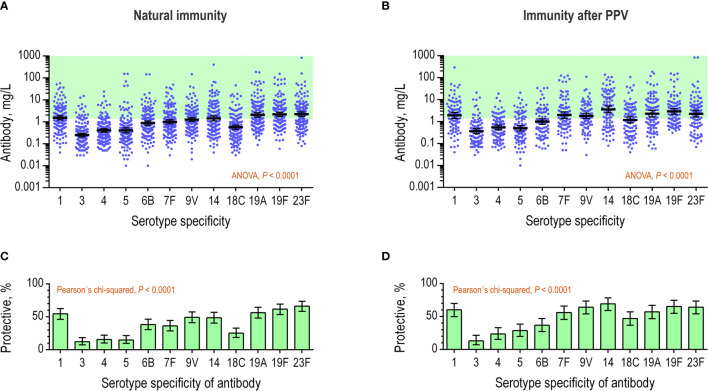
Serotype-specific antibody levels in the patient serum samples. The concentrations (mg/L) of 12 different antibodies were determined in serum samples by a multiplex, bead-based assay. **(A)** The natural immunity of the patients (*n* = 154) displayed for each of the antibodies as continuous variables. The green area indicates concentrations of at least 1.3 mg/L, which is considered as ‘protective’ under AAAAI guidelines. Error bars are geometrical means with 95% confidence intervals. The antibodies were tested for different levels using repeated-measures ANOVA. **(B)** Data from previous panel showing the proportion of patients with antibody levels of at least 1.3 mg/L (i.e., ‘protective level’) for each specific antibody. **(C)** As in the panel A, but for levels measured in serum samples collected after PPV vaccination (*n* = 98). **(D)** Data from previous panel, showing the proportion of patients with antibody levels of at least 1.3 mg/L for each specific antibody.

The natural immunity (pre-existing antibody levels) could be assessed for 154 of the patients. This included data from assays originally requested as qualitative. We found that the antibody levels differed markedly between different serotype-specificities (**Figure 4A**). The mean level differed approximately ten-fold between anti-serotype 3 antibody (0.25 mg/L) and anti-serotype 23F antibody (2.3 mg/L). Anti-serotype 4 antibody showed the least variation between patients (480-fold) and anti-serotype 5 antibody showed the greatest variation (15,000-fold). The levels of the 12 antibody specificities correlated positively in the patients, e.g., the correlations between anti-serotype 4 antibody levels and the levels of each of the other 11 antibodies displayed Spearman’s ρ of minimum 0.30 (*p* ≤ 0.0001). As expected, the proportion of patients with levels of at least 1.3 mg/L differed markedly between the different serotype-specific antibodies (**Figure 4B**). The proportion differed as much as five-fold between anti-serotype 3 antibody (12%) and anti-serotype 23F antibody (66%).

For assessment of the immunity after PPV vaccination, antibody quantifications were available for 98 patients. The follow-up time was between four and eight weeks for 72% of the patients (median 5 weeks), shorter for 6.5% (median 3 weeks), and longer for 22% (median 19 weeks). We found that the mean antibody levels differed markedly for the different serotype specificities after vaccination (**Figure 4C**). The mean level differed approximately 10-fold between anti-serotype 3 antibody (0.37 mg/L) and anti-serotype 14 antibody (3.6 mg/L). Anti-serotype 4 antibody showed least variation between patients (380-fold) and anti-serotype 23F antibody showed most variation (12,000-fold). The antibody levels of the 12 specificities correlated positively in the individual patients, e.g., the correlations between anti-serotype 4 antibody and each of the other 11 antibody specificities displayed Spearman’s ρ of minimum 0.30 (*p* ≤ 0.0029). Again, the proportion of patients with antibody levels of at least 1.3 mg/L differed markedly between the different serotype-specificities (**Figure 4D**). The proportion differed as much as 5-fold between anti-serotype 3 antibody (13%) and anti-serotype 14 antibody (69%).

The collective results confirm that concentrations differ between different serotype-specific antibodies in patients with suspected primary immunodeficiency diseases. In addition, serotype-specific antibodies show different probabilities for fulfilling the criterion for a protective level as defined in the AAAAI guidelines.

### Dichotomous Assessment of Diagnostic Vaccination Is Not Robust

We explored how the different probabilities of reaching 1.3 mg/L among different serotype-specific antibodies affected the outcome of diagnostic vaccination based on the AAAAI guidelines. Specifically, we separated the 12 measured serotype-specific antibodies into different arbitrary antibody panels (I, II, III, and IV) and compared the outcomes. The four panels each contained measurements of six different antibody specificities to eliminate the effect of different antibody numbers (**Figure 1B**). Partial combinations of the measured antibody specificities were used for this particular sensitivity analysis only. In all other analyses, all antibody specificities measured were applied. For this analysis, we used the available antibody measurements obtained after PPV vaccination (*n* = 98).

**Figure 5:**
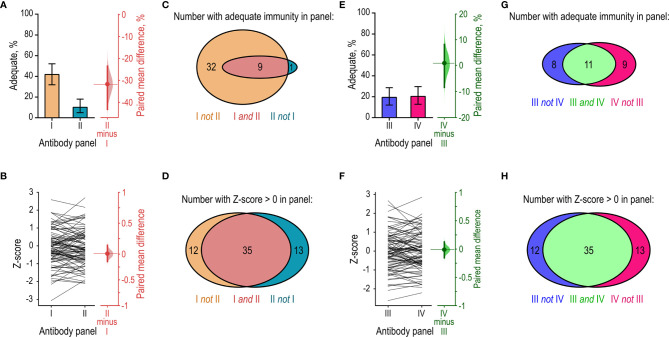
Sensitivity-analysis of interpretation using the AAAAI guidelines and Z-scores. The same cohort of patients (*n* = 98) were assessed for adequate immunity after PPV vaccination using different panels of serotype-specific antibodies for the interpretation. Each panel contained six out of the 12 quantified serotype-specific antibodies. **(A)** Left: The proportions of patients considered to have achieved ‘adequate immunity’ in each of two antibody panels (I and II) according to the AAAAI guidelines. Right: The paired mean difference in proportions (dot) with 95% confidence interval (vertical error lines) and bootstrap sampling distribution (normalized histogram). Panel I contained the six serotype-specific antibodies that most frequently had levels of 1.3 mg/L or higher in the patients. Panel II contained the six serotype-specific antibodies that least frequently had levels of 1.3 mg/L or higher in the patients. **(B)** Left: Paired Z-scores for the individual patients calculated using data from panel I and panel II. Right: The paired mean difference in Z-score from the two panels. **(C)** Venn-diagram showing the number of patients with adequate immunity in panels I and II under AAAAI guidelines. Panels I and II identified an unequal numbers of the patients, and the results showed little overlap. **(D)** As in **(C)**, but for Z-scores. Panels I and II now identified comparable numbers of the patients, and the results largely overlapped. **(E)** As in **(A)**, but for two other panels (III and IV). These panels were composed to provide an equal proportion of patients with ‘adequate immunity’ under AAAAI guidelines. **(F)** Comparison of the Z-scores calculated for the patients using the data from panels III and IV. **(G)** Venn-diagram showing the number of patients with adequate immunity in panels III and IV according to the AAAAI guidelines. Panels III and IV identified comparable numbers of the patients, but the results showed little overlap. **(H)** As in **(G)**, but for Z-scores. Panels III and IV identified comparable numbers of the patients, and the results largely overlapped.

We first examined a worst-case scenario by comparing the outcomes of two antibody panels (I and II). Panel I contained the six antibody specificities with the highest proportion of levels at or above 1.3 mg/L (i.e., anti-serotype 14, -19F, -9V, -23F, -1, and -19A). Panel II contained the six antibodies with the lowest proportion of levels at or above 1.3 mg/L (anti-serotype 7F, -18C, -6B, -5, -4, and -3). Analysis using panel I resulted in ‘adequate immunity’ for 42% of the patients and analysis using panel II resulted in ‘adequate immunity’ for only 10% of the patients (paired mean difference 32% [-43%; -24%]) (**Figure 5A**). The arbitrary choice of serotype-specific antibodies in the two panels thus resulted in a four-fold difference in the proportion of patients with adequate immunity under AAAAI guidelines. Next, we made a recalculation based on the Z-score approach. The Z-score is theoretically more robust across interpretation of different serotype-specific antibodies (10). To test this in practice, we compared the mean Z-scores for panel I and II. As anticipated, no systematic difference in Z-score was found when using either panel (paired mean difference 0.0 [-0.12; 0.14]) (**Figure 5B**). Consistency of the outcome is also important. According to the AAAAI guidelines, 41 patients had adequate immunity in panel I but only nine of these patients (22% [9.8%; 34%]) also displayed adequate immunity in panel II (**Figure 5C**). Ten patients had adequate immunity in panel II and nine of these patients (90% [50%; 100%]) also showed adequate immunity in panel I (**Figure 5C**). For the Z-score, the limit for adequate immunity is not yet defined. To facilitate a comparison of Z-score consistency between antibody panels, we assigned an arbitrary cutoff of 0. Forty-seven patients had Z-score above 0 in panel I and 35 of these patients (76% [59%; 84%]) also had Z-score above 0 in panel II (**Figure 5D**). The reverse comparison gave similar results (**Figure 5D**). Thus, in the worst-case scenario, interpretation based on Z-score was markedly less sensitive to the choice of serotype-specific antibodies than the AAAAI guidelines.

We then examined a best-case scenario by constructing two other antibody panels (III and IV) of similar propensity for achieving ‘adequate immunity’ under the AAAAI guidelines. To design these panels, we ranked the twelve serotype-specific antibodies according to their frequency of being at least 1.3 mg/L. Panel III contained the six antibodies with rank numbers 1, 4, 5, 8, 9, and 12 (i.e., anti-serotype 14, -23F, -1, -18C, -6B, and -3). Panel IV contained the six antibodies with rank numbers 2, 3, 6, 7, 10, and 11 (i.e., anti-serotype 19F, -9V, -19A, -7F, -5, and -4). As intended, a similar proportion of the patients had adequate immunity according to the AAAAI guidelines in the two panels (**Figure 5E**). Z-scores were also similar (**Figure 5F**). According to the AAAAI guidelines, 19 patients had adequate immunity in panel III but only 11 of these patients (58% [26%; 74%]) also had adequate immunity in panel IV (**Figure 5G**). Twenty patients had adequate immunity in panel IV but only 11 of these patients (55% [25%; 70%]) also had adequate immunity in panel III (**Figure 5G**). However, compared to interpretation according to the AAAAI guidelines, significantly better consistency was achieved when data from panels III and IV were interpreted with Z-scores (**Figure 5H**). With the Z-scores, the inconsistency between panels III and IV corresponded to that observed between panels I and II (cf. **Figures 5D, H**).

The collective results support the conclusion that the Z-score provides more robust results than the AAAAI guidelines.

### Natural Immunity to Pneumococci in Patient Subgroups

Next, we compared the two approaches for interpretation in different patient subgroups. The subgroups were defined according to infection profile, gender, and age groups. All measured serotype-specific antibodies were included in a single panel in this part of the study. First, we examined the natural immunity in all patients (*n* = 154). Overall, 12% [7.6%; 19%] had adequate immunity according to the AAAAI guidelines.

**Figure 6:**
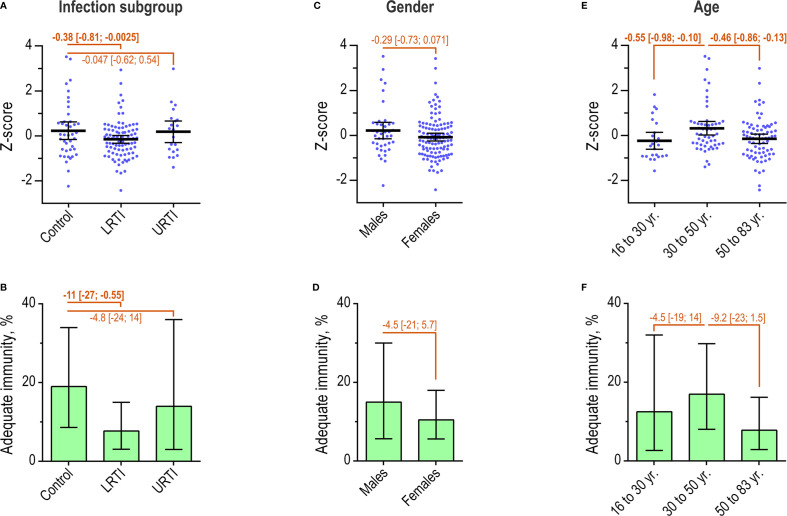
Natural immunity in patient subgroups. **(A)** Individual Z-scores and mean with 95% confidence interval for patients (*n* = 154), by infection profiles. Differences between groups were determined as indicated. **(B)** The percentage of patients with ‘adequate’ immunity according to the AAAAI guidelines, by infection profiles. Error bars are 95% confidence intervals. **(C)** As in A, but for patients by gender. **(D)** As in B, but for patients by gender. **(E)** Individual Z-scores for patients by age intervals. **(F)** As in B, but for patients by age group.

When divided by infection profile, the control group (*n* = 42) displayed higher mean Z-score than patients in the LRTI group (*n* = 91) (difference -0.38 [-0.81, -0.0025]) (**Figure 6A**). No significant difference was observed between the control group and the URTI group (*n* = 21). The dichotomous approach also identified more frequent adequate immunity in the control group, 19%, compared with patients in the LRTI group, 7.7% (difference -11% [-27%; -0.55%]) (**Figure 6B**). Similarly, this approach did not identify a significant difference between patients in the control group and the URTI group.

Neither of the two approaches identified any difference according to gender (**Figures 6C, D**).

The relationship between age and Z-score showed an inverted, flattened U-shaped relationship, with Z-scores peaking in the age-group 30 to 50 years (**Figure S2**). Compared with patients in the age-group 30 to 50 years (*n* = 53), younger patients (*n* = 24) and older patients (*n* = 77) had lower mean Z-score (difference -0.55 [-0.98; -0.097] and -0.46 [-0.86, -0.13]) (**Figure 6E**). Adequate immunity according to the AAAAI guidelines also peaked in the age-group 30 to 50 years (**Figure S3**). However, the dichotomous approach did not detect statistical differences relating to age groups (**Figure 6F**).

In summary, the estimated natural immunity was weaker in patients with recurrent LRTI compared with the control group. The Z-score, but not the AAAAI guidelines, identified stronger natural immunity in patients aged 30 to 50 years compared with both younger and older patients. Gender was not associated with any difference in natural immunity.

### Immunity After PPV Vaccination in Patient Subgroups

We compared the outcomes of the two approaches for interpreting immunity after PPV vaccination in patient subgroups (*n* = 98). Overall, 23% [15%; 33%] had adequate immunity based on the AAAAI guideline principles.

**Figure 7:**
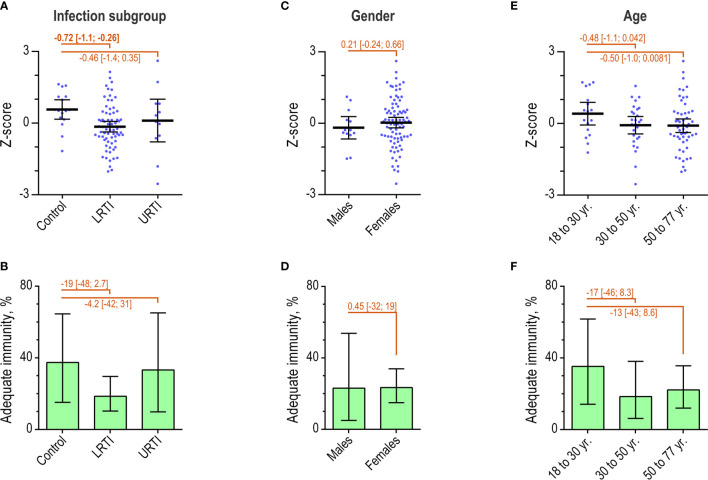
Immunity after PPV in patient subgroups. **(A)** Individual Z-scores and mean with 95% confidence interval for patients (*n* = 98) by infection profiles. Differences between groups were determined as indicated. **(B)** The percentage of patients with ‘adequate’ immunity according to the AAAAI guidelines, by infection profiles. Error bars are 95% confidence intervals. **(C)** As in A, but for patients by gender. **(D)** As in B, but for patients by gender. **(E)** Individual Z-scores for patients by age intervals. **(F)** As in B, but for patients by age group.

When divided according to infection profile, the control group (*n* = 16) had a higher mean Z-score than the LRTI group (*n* = 70) (difference -0.72 [-1.1; -0.25]) (**Figure 7A**). No significant difference was observed between the control group and the URTI group (*n* = 12). However, interpretation with the dichotomous approach did not detect significant differences relating to infection subgroups (**Figure 7B**).

Neither approach for interpretation identified any significant differences relating to gender or age groups (**Figures 7C–F**). Increasing age did, however, show a tendency towards decreasing immunity (**Figures S4, S5**).

In contrast to interpretation under AAAAI guidelines, the Z-score identified weaker immunity after PPV in the LRTI patient group compared with the control group. None of the approaches for interpretation detected significant differences in immunity related to gender or age.

## Discussion

This study demonstrates two significant caveats in diagnostic vaccination based on the AAAAI guidelines. First, correct procedure may be difficult to achieve in routine clinical settings. Second, the dichotomous principle applied for result interpretation introduces complex effects of two arbitrary choices, namely the number and the serotype-specificity of the quantified antibody levels. In contrast, evaluation based on continuous variables, such as Z-scores, may simplify the procedure and add robustness. In our study, Z-scores after PPV vaccination discriminated patients with recurrent lower respiratory tract infections from patients with infections that did not evoke suspicion of antibody deficiency. The AAAAI guidelines failed in detecting this difference.

The retrospective design of this study is a strength in providing unbiased data for our audit of diagnostic vaccination in routine clinical practice. A monitored clinical trial likely offers less valid data for an audit. A further asset of the study is that a single laboratory performed all antibody measurements using the same assay, which adds comparability to the data set. The use of a single statistical approach for determining effect sizes for the dichotomous variable and the continuous variable also aids comparability.

This study had limitations. The interpretations made by the individual clinicians were not studied, mainly because clear statements on these matters were rarely provided in the medical records. We therefore cannot rule out that erroneous data interpretation by the clinicians may have reduced the actual frequency of correct procedures to less than the 17% we report. We did not include intentional deviations from the guidelines in our audit. For instance, it is local practice to refrain from diagnostic vaccination of the rare patients with very low plasma IgG concentration (below 1–2 g/L) and symptoms prompting immediate IgG replacement therapy. Also, baseline antibody level measurements are required under AAAAI guideline but were omitted for several patients undergoing diagnostic vaccination. In most cases, this was likely a deliberate choice, insofar as the local clinicians find the absolute antibody concentrations after vaccination of direct interest. The study design and available data do not allow confident conclusions on the underlying cause of the high rate of failure. We do, however, suspect that the complexity of the AAAAI guidelines is responsible. Data on other laboratory parameters, such as the levels of total IgG and IgG subclasses, were not included. We find that such data would not contribute to this head-to-head comparison of methods for assaying diagnostic vaccination. Others have reported a lack of association between these parameters and the outcome of diagnostic vaccination (21). We find that the number of patients included in the study, although limited, is sufficient to assess the applicability of a diagnostic test intended for use in the evaluation of individual patients.

We found it relevant to examine the actual conductance of diagnostic vaccination. Meticulous guidelines are of little use if they are virtually impossible to comply with in clinical practice. Strict interpretation in agreement with recommendations of the AAAAI guidelines was possible in only one out of six patients. Our design does not allow us to infer that this is a general trend. But we see no reason to suspect that our observed compliance is especially poor compared with that of other centers. Indeed, there are indications that the procedure also fails frequently elsewhere. For example, Barton and coworkers reported that for 14 out of their 18 patients with IgG2 subclass deficiency, historical data were insufficient to interpret diagnostic vaccination under AAAAI guidelines (22). We suspect that the complexity of the AAAAI guidelines is responsible for the high failure frequency.

Based on the AAAAI criteria, adequate immunity was present in 23% of our patients after vaccination. This is low compared with the frequency reported for some cohorts [typically at least 50% (15, 23–25)] although some studies report similar results (26, 27). Several factors may explain the different findings. The AAAAI guidelines are inherently unreliable for comparing cohorts tested with different panels of serotype-specific antibodies (**Figures 1B**, **5**) (10). Also, use of different assays for antibody quantification is problematic because of poor inter-assay comparability (28–31). Cohorts are likely to differ in their ability to respond to vaccination. Our cohort, comprised of patients referred to advanced laboratory tests for primary immunodeficiency diseases, may be less capable of producing specific antibodies than the majority of reported cohorts.

Both the natural immunity and the immunity after PPV are reported as lower in adults with recurrent lower respiratory tract infections compared with healthy controls (32). In support of such reports, we found that assessment using the AAAAI principles as well as Z-scores identified lower natural immunity in the LRTI group compared with the patient controls suffering from infections that do not indicate antibody deficiency. However, only interpretation based on Z-scores revealed lower immunity after PPV in the LRTI group compared with the patient controls, whereas interpretation with the AAAAI guidelines failed to demonstrate this difference. Estimates based on Z-scores thus seem more sensitive for detection of differences in antibody immunity between patient groups.

Recurrent URTI may also be a sign of antibody deficiency. However, neither approach detected lower immunity in such patients. Our study included few patients with URTI (*n* = 12 for assessment of immunity after PPV vaccination) and therefore has low statistical power for assessing this issue. We therefore cannot rule out lower immunity in patients with recurrent URTI.

Z-scores detected higher natural immunity in patients aged 30 to 50 years compared with younger as well as older patients. This was not detected by the dichotomous assessment. We speculate that the lower immunity is explained by fewer previous natural immunizing events in the younger patients and by waning immunity in the older patients, in agreement with the general view in the field (33).

We anticipate that adopting continuous variables for assaying diagnostic vaccination can improve interpretation of diagnostic vaccination. The change will also allow better comparison of different cohorts, especially when different numbers of antibodies and different serotype-specific antibodies are tested. Interpretation based on dichotomous principles is inherently sensitive to differences in these factors (see *Introduction* and **Figure 1B**), whereas interpretation based on continuous interpretation is more robust (10). Another shortcoming of the dichotomous principles is that the individual antibody measurement is reduced to an “all-or-nothing” outcome, which reduces information and over-emphasizes trivial differences in concentrations near the cut-off. For example, the difference between an antibody concentration of 1.3 mg/L and 1.2 mg/L is unlikely to be of clinical relevance, yet one is deemed protective whereas the other is not. The Z-score is thus more robust to interpretation under different antibody panels than dichotomous outcomes based on the AAAAI guidelines (**Figure 5**). Moreover, we expect that Z-scores will provide more consistent results across laboratories than the AAAAI guidelines, even when the same antibody specificities are tested. Different laboratories may estimate the concentration of a given antibody specificity very differently (28–31), which is a strong disadvantage for interpretation with the AAAAI guidelines. Such inter-laboratory differences are less critical for Z-scores, as long as the concentration estimates show good correlations. This should be examined in future studies.

To simplify the practical procedure, we propose to omit antibody quantification before vaccination and limit the future protocol to the following:

1. PPV vaccination of the patient.
2. Follow-up blood sample after four to eight weeks only.
3. Quantification of the levels of individual serotype-specific antibodies.
4. Calculation of the patient Z-score by the laboratory.
5. Data evaluation.

The protocol can be used with other polyvalent vaccines and for multiple monovalent vaccines that are administered simultaneously. The proposed four to eight weeks interval for blood sampling simply complies with the AAAAI recommendations for diagnostic vaccination with PPV. This recommendation does not appear supported by data (7), suggesting that the timing may potentially be optimized. We propose to quantify at least six different serotype-specific antibodies, based on previous findings on the relationship between result variations and the number of tested antibodies with the Z-score (10). The calculation of Z-scores requires data on the antibody levels in a suitable reference population such as healthy persons. Z-scores of patients can be interpreted relative to the fraction of the reference persons with equal or lower Z-scores. The estimations may apply the probability density function for the standard normal distribution or a non-parametrical approach.

Although promising, the Z-score approach is not yet ready for clinical application, but requires further study. The suggested protocol should thus be tested and optimized further based on the findings in different patient cohorts and by different laboratories. We plan a retrospective study of the proposed protocol, which will include patients referred to our institution from the end of the inclusion for the present study (August 2016) to the present day. However, prospective studies of the Z-score approach are highly desirable before possible dissemination into clinical practice.

Entirely different approaches for assessing the antibody competence of patients may also be of clinical interest. We recently reported that the level of naturally occurring antibodies against terminal galactose-*α*-1,3-galactose (anti-αGal) predicts the outcome of diagnostic vaccination in HIV infected adults (34). Anti-αGal antibodies are of particular interest in patients with suspected antibody deficiency. The level of anti-αGal antibodies is low in such patients (17, 35, 36). In humans, the anti-αGal antibodies seem important by targeting various common pathogens (17, 37), leading to activation of immunological effector mechanisms (17, 38), and ultimately protection (17). Future studies may therefore examine the association between the anti-αGal antibodies and vaccine response in patients with suspected primary antibody deficiency.

In conclusion, patients may benefit from revised protocols for the conductance and interpretation of diagnostic vaccination. We provide evidence suggesting that the AAAAI guidelines for diagnostic vaccination are difficult to apply in clinical practice. Even when executed in accordance with guidelines, the categorical interpretation of results remains problematic. We therefore propose that a more pertinent evaluation is achievable with Z-scores, which may also simplify the procedure of diagnostic vaccination.

## Data Availability Statement

The original contributions presented in the study are included in the article/**Supplementary Material**. Further inquiries can be directed to the corresponding author.

## Ethics Statement

The studies involving human participants were reviewed and approved by The Ethics Committee in Central Denmark Region Regionssekretariatet Juridisk Kontor Skottenborg 26 8800 Viborg Denmark. Written informed consent from the participants’ legal guardian/next of kin was not required to participate in this study in accordance with the national legislation and the institutional requirements.

## Author Contributions

AH and JB conceived the study. AS, CL, and CJ provided data. JB performed data analyses. CJ and CL provided intellectual inputs. AH, MP, and JB wrote the manuscript. All authors edited and approved the manuscript. All authors contributed to the article and approved the submitted version.

## Funding

This work was funded by the public healthcare system of Denmark.

## Conflict of Interest

The authors declare that the research was conducted in the absence of any commercial or financial relationships that could be construed as a potential conflict of interest.

## Publisher’s Note

All claims expressed in this article are solely those of the authors and do not necessarily represent those of their affiliated organizations, or those of the publisher, the editors and the reviewers. Any product that may be evaluated in this article, or claim that may be made by its manufacturer, is not guaranteed or endorsed by the publisher.
