# Supplementary material for: Diagnostic Vaccination in Clinical Practice
Source: Front Immunol. 2021 Sep 30;12:717873. doi: 10.3389/fimmu.2021.717873 (PMC8514775; doi:10.3389/fimmu.2021.717873)
Supplement: Supplementary file 1 [file DataSheet_1.docx]

Supplementary Material


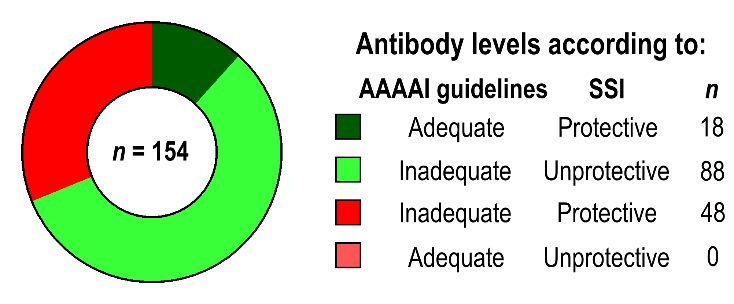


**SUPPLEMENTARY FIGURE 1.** Comparison of the qualitative conclusions achieved for the antibody levels using interpretation according to the AAAAI guidelines and the laboratory that performed the measurements (SSI). The measured antibody levels in 154 different samples were used (the samples used to study natural immunity). Twelve serotype-specific antibodies were quantified in each sample. The expert guideline defines the antibody levels in a sample as adequate when at least 70% of measured serotype-specific antibodies are 1.3 mg/L or higher (1, 2). SSI defines protective immunity as a geometrical mean of the measured serotype-specific antibodies of at least 1.0 mg/L. The pie-chart shows the part of samples with agreement between the two approaches in green colors and disagreement in red color. As can be observed from the figure, the qualitative assessment performed by SSI will markedly overestimate the part with adequate or protective antibody levels as compared to interpretation according to the AAAAI guidelines.


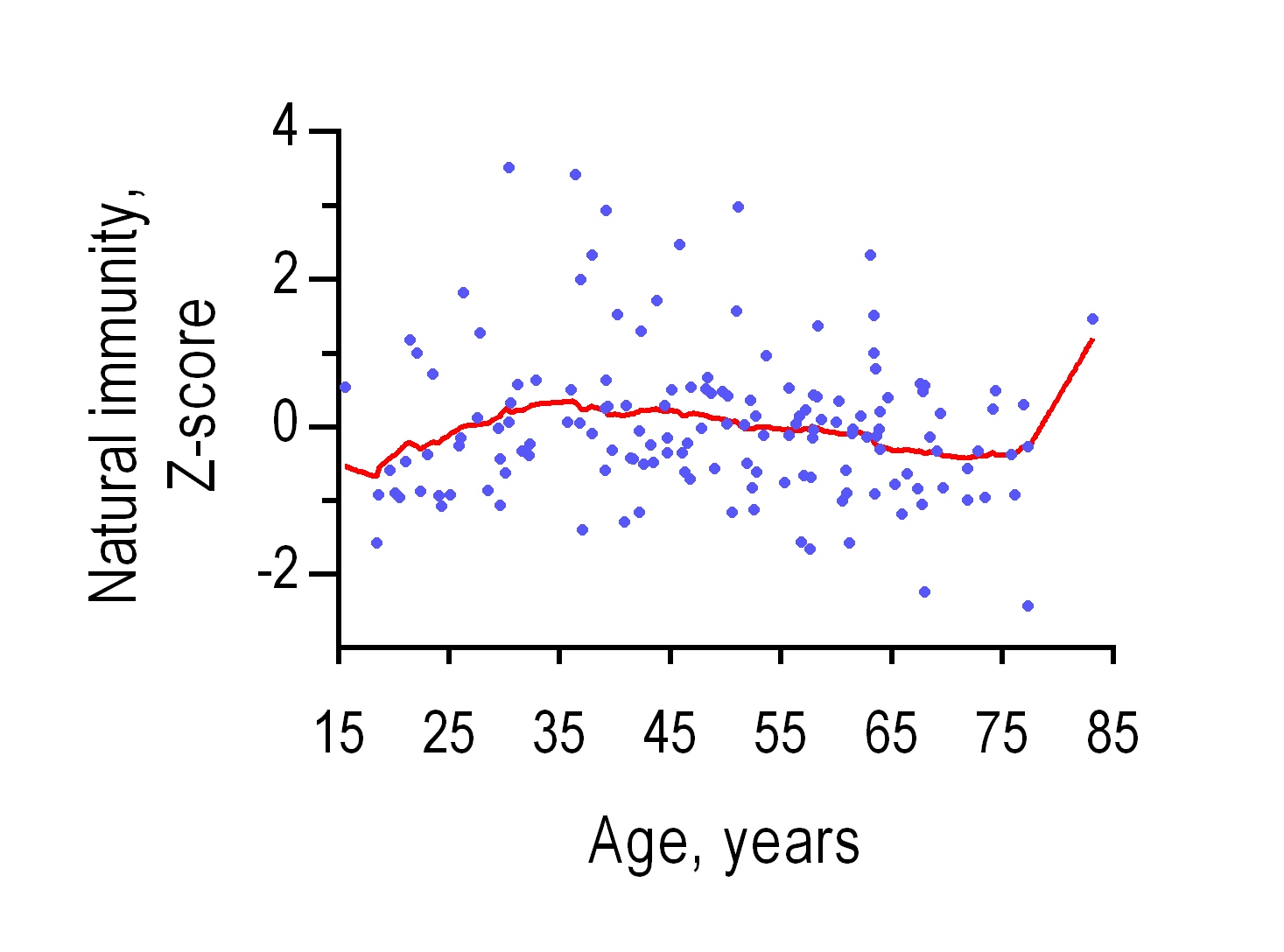


**SUPPLEMENTARY FIGURE 2.** Relationship between the Z-scores of natural immunity and age in the cohort. The red line is the Locally Weighted Scatterplot Smoothing (LOWESS) curve with five points per smoothing window.


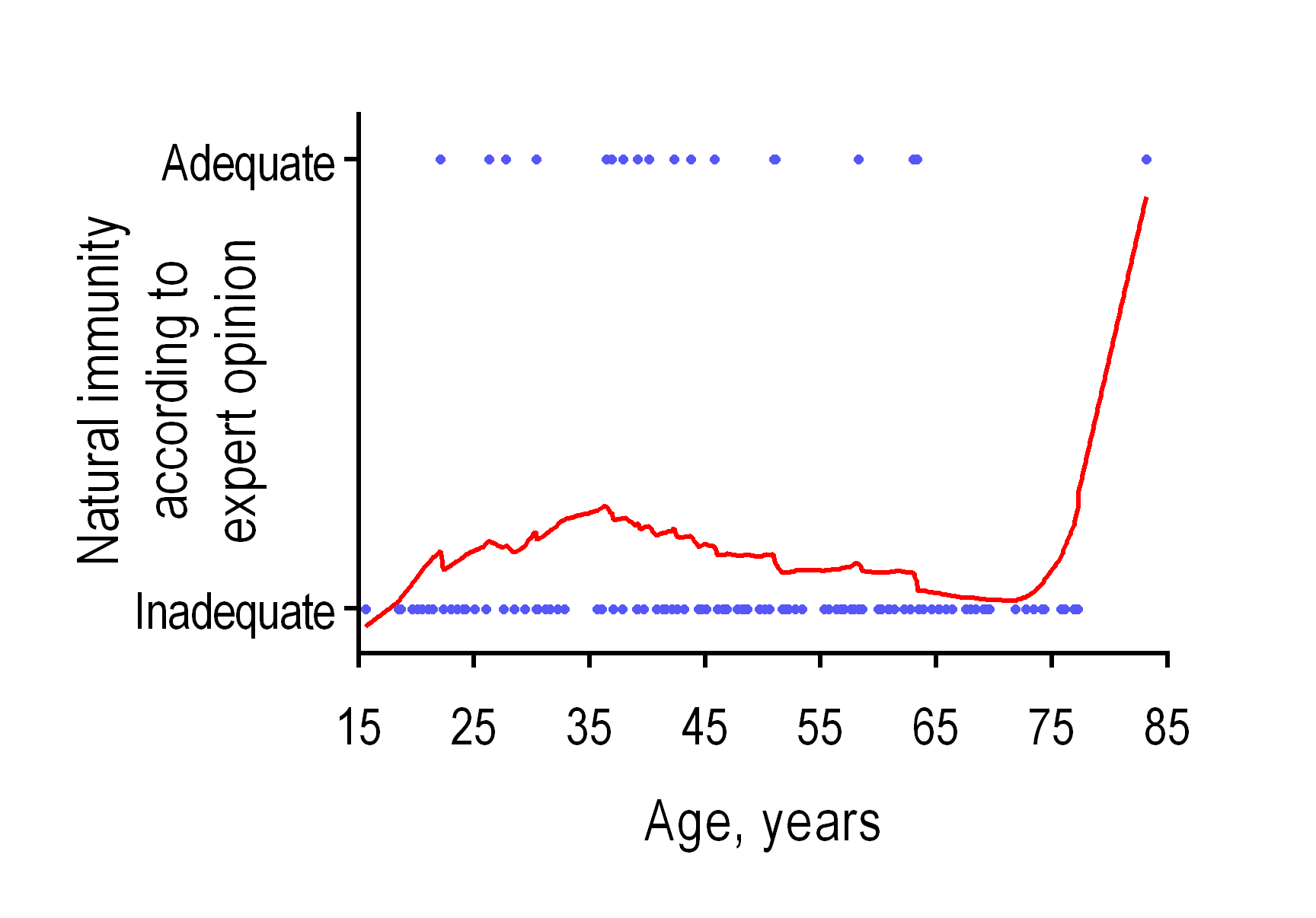


**SUPPLEMENTARY FIGURE 3.** Relationship between the natural immunity, assessed according to expert guidelines, and age in the cohort. The red line is the LOWESS curve with five points per smoothing window.


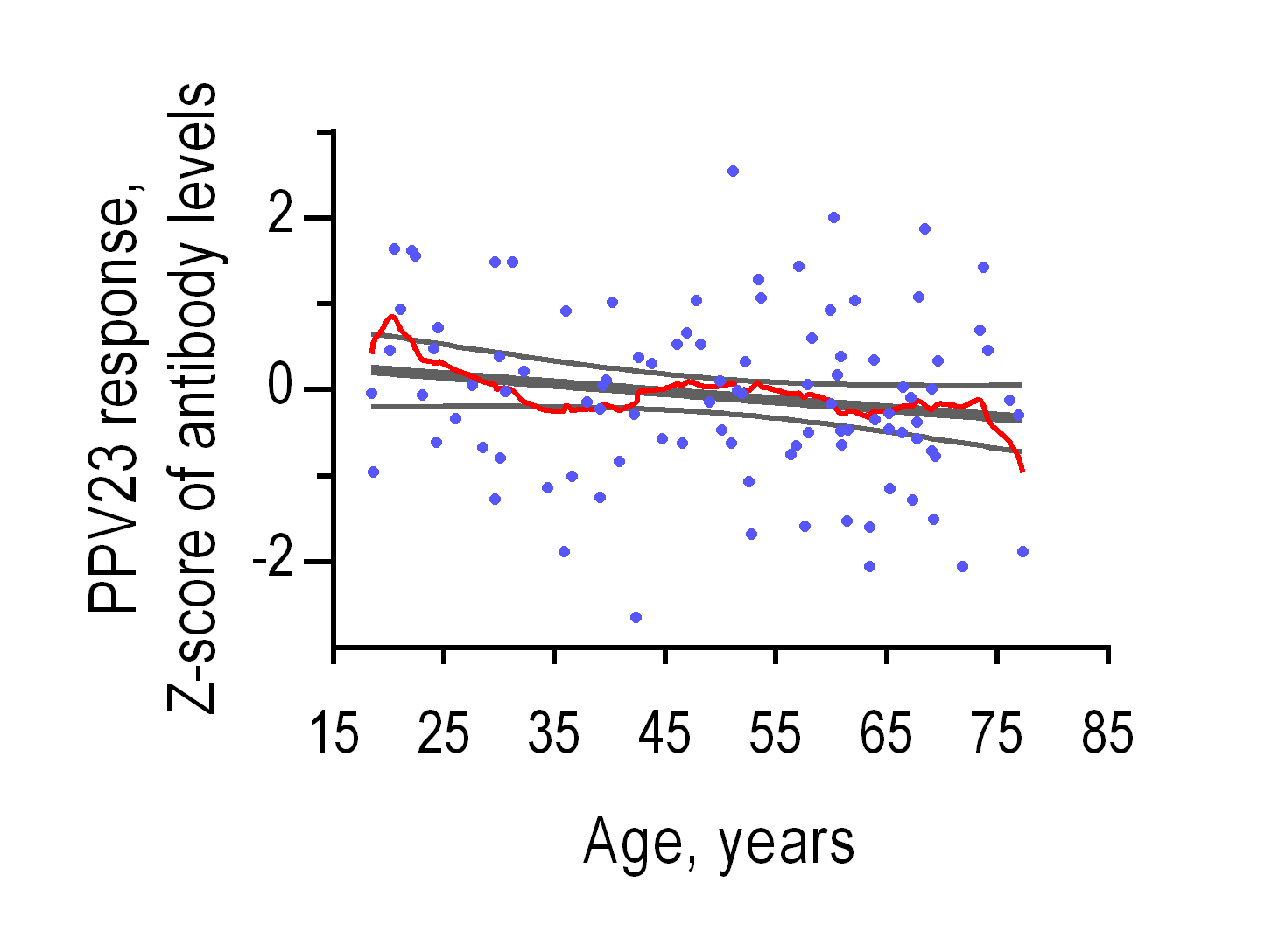


**SUPPLEMENTARY FIGURE 4.** Relationship between the Z-scores of PPV23 responses, based on antibody levels, and age in the cohort. The red line is the LOWESS curve with five points per smoothing window. The grey lines are the linear regression curve with 95% confidence intervals. The slope estimate is -0.0096 [-0.022, 0.00259]/year.


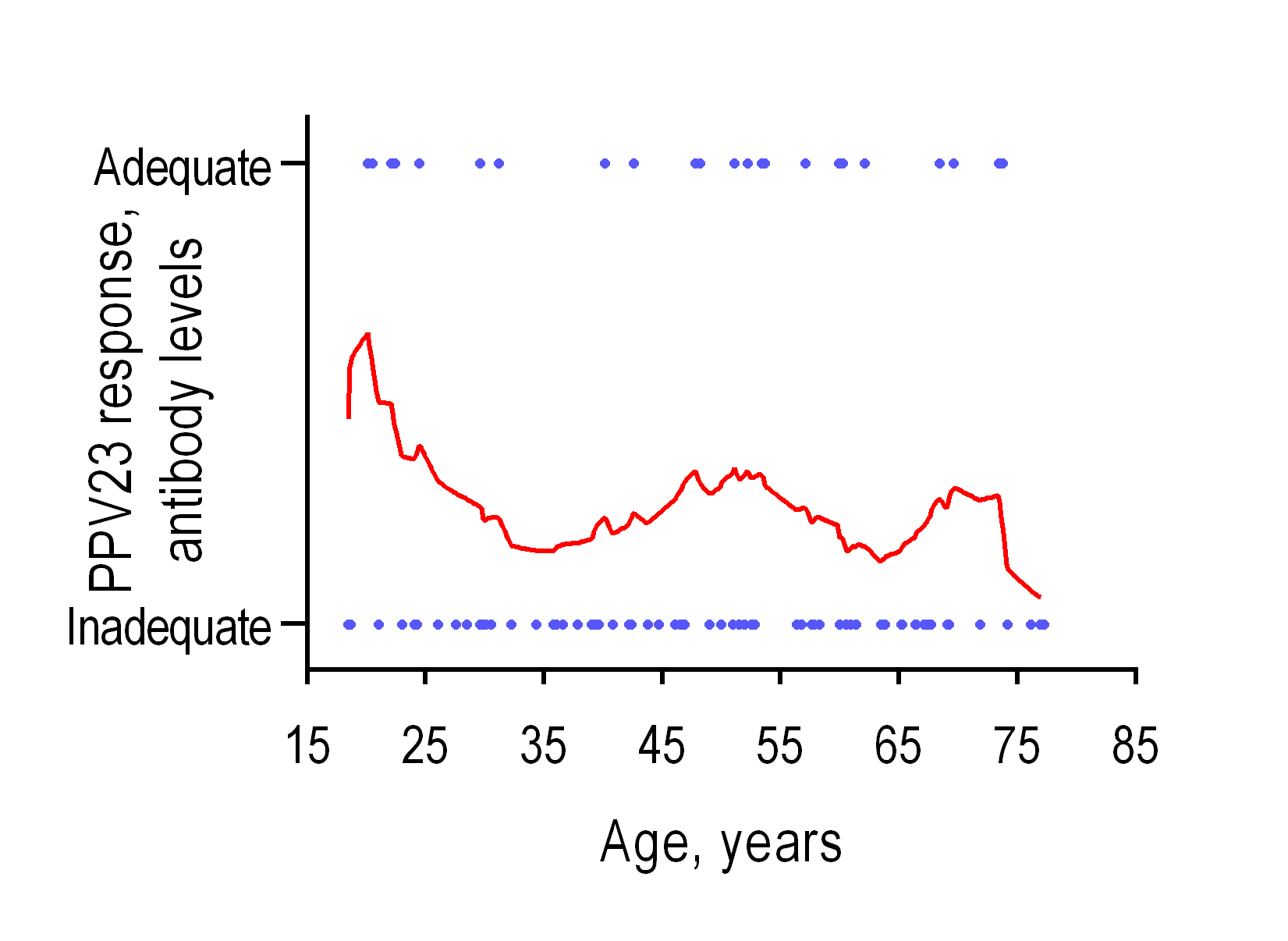


**SUPPLEMENTARY FIGURE 5.** Relationship between the PPV23 response, based on antibody levels, according to expert opinion and age in the cohort. The red line is the LOWESS curve with five points per smoothing window.

**REFERENCES**

1. Orange JS, Ballow M, Stiehm ER, Ballas ZK, Chinen J, De La Morena M, et al. Use and interpretation of diagnostic vaccination in primary immunodeficiency: a working group report of the Basic and Clinical Immunology Interest Section of the American Academy of Allergy, Asthma & Immunology. *The Journal of allergy and clinical immunology.* 2012;130(3 Suppl):S1-24.

2. Bonilla FA, Khan DA, Ballas ZK, Chinen J, Frank MM, Hsu JT, et al. Practice parameter for the diagnosis and management of primary immunodeficiency. *The Journal of allergy and clinical immunology.* 2015;136(5):1186-205.e1-78.
